# Clinical Effectiveness and Magnetic Resonance Imaging-Based Endurability of Matrix-Associated Autologous Chondrocyte Implantation with an Autologous Periosteal Flap for Articular Cartilage Defects of the Knee Joint

**DOI:** 10.3390/jcm15093445

**Published:** 2026-04-30

**Authors:** Taku Tadenuma, Yuji Uchio, Takuya Wakatsuki, Hiroshi Takuwa, Suguru Kuwata

**Affiliations:** Department of Orthopaedic Surgery, Faculty of Medicine, Shimane University, 89-1 Enya-cho, Izumo 693-8501, Shimane, Japan; uchio@med.shimane-u.ac.jp (Y.U.); waka87@med.shimane-u.ac.jp (T.W.); h.takuwa@med.shimane-u.ac.jp (H.T.); kuwata@med.shimane-u.ac.jp (S.K.)

**Keywords:** cartilage injury, matrix-associated autologous chondrocyte implantation, T1*ρ*, T2 map, magnetic resonance observation of cartilage repair tissue

## Abstract

**Objectives**: To evaluate the effectiveness and durability of matrix-associated autologous chondrocyte implantation with periosteal flap (pMACI) in treating knee cartilage defects using clinical scores and MRI evaluations. **Methods**: Data were collected from 37 knees of 17 patients, with a mean follow-up of 5 years (range: 0.1–20 years). Clinical outcomes were assessed using the Lysholm Knee Scoring Scale (LKS) and Knee Injury and Osteoarthritis Outcome Score (KOOS). Tissue quality was quantitatively evaluated using MRI T1*ρ* and T2 mapping (biochemical) and MR observation of cartilage repair tissue: MOCART 2.0 (morphological). A linear mixed model was used to identify factors affecting outcomes, including etiology (trauma, OCD, OA), graft site, and defect size. **Results**: At the 20-year follow-up, clinical scores remained significantly improved from baseline (mean LKS: 55.6 to 86.5; KOOS: 37.8 to 70.8). The biochemical MRI parameters (T1*ρ* and T2 values) stabilized at levels comparable to native cartilage across all etiologies and sites (*p* = 0.326 and 0.412, respectively), indicating stable long-term tissue quality. In contrast, the MOCART 2.0 scores significantly declined over time (annual rate: −1.14 points; *p* < 0.001). Etiology was a significant factor; the OA group showed significantly lower clinical and MOCART scores compared to the trauma/OCD groups (*p* < 0.05). However, no significant differences were found in LKS and KOOS based on graft site (*p* = 0.489) or defect size (*p* > 0.05). **Conclusions**: pMACI may be a highly durable treatment capable of maintaining biological tissue quality and providing clinical benefits for two decades. The observed morphological deterioration after 20 years likely reflects joint-wide aging—especially in OA cases—rather than graft failure, highlighting the importance of long-term MRI monitoring.

## 1. Introduction

Articular cartilage is a hyaline cartilage characterized by its unique structure: it lacks nerves, blood, and lymphatic vessels, contains few cells, and has an abundant extracellular matrix. This is key to maintaining joint function with painlessness, low friction, elasticity, and durability. However, this feature can hinder repair [1]. Examinations of over 30,000 arthroscopic procedures showed that approximately 60% of patients had high-grade cartilaginous defects, with lesion depths affecting 50% or more of the cartilage surface [2,3]. Increased contact stress in adjacent healthy cartilage may lead to degeneration and progression of osteoarthritis (OA) [4]. Post-traumatic OA accounts for approximately 12% of all OA cases [5]. Previous cartilage repair attempts did not aim to restore hyaline cartilage; therefore, while short-term outcomes such as pain reduction and improved mobility were favorable, the results deteriorated over time. However, regenerative medicine has recently been applied to repair the hyaline cartilage [6].

Brittberg et al. were the first in the world to develop autologous cultured chondrocyte implantation (ACI), involving the injection of autologous chondrocytes, expanded through monolayer cultivation, into the defect, which is covered with an autologous periosteal flap from the tibia (pACI) [7]. While studies have demonstrated the effectiveness and safety of pACI in intermediate- and long-term follow-ups [8,9,10,11,12,13,14,15,16], concerns remain regarding the maintenance of the chondrocyte phenotype during cultivation, cell leakage from the defect, and an uneven cell distribution during implantation [9,11,12,17,18,19,20,21,22,23,24]. Ochi et al. developed matrix-associated autologous chondrocyte implantation (MACI) covered with an autologous periosteum (pMACI), using chondrocytes cultured ex vivo for 4 weeks in an atelocollagen gel, and then transplanted [25,26]. Studies have shown that pMACI effectively addresses these issues [27,28]. Clinical trials have reported favorable clinical and MRI outcomes for up to 6 years post-implantation, with minimal adverse events [29,30,31,32]. A real-world data analysis over 2 years in Japan showed that pMACI improved outcomes in approximately 75% of patients [33]. From a biological standpoint, the efficacy of the periosteal cover in pMACI can be elucidated through the “epiligament theory” [34,35,36]. Similarly to the epiligament, which functions as a crucial reservoir for vasculature and progenitor cells necessary for ligament repair [34], the autologous periosteum may serve as a functional biological source. This may facilitate the recruitment of regenerative cells and growth factors, thereby supporting the long-term integration of atelocollagen-embedded chondrocytes.

Despite these successes, concerns persist regarding complications, with approximately one-third of patients experiencing adverse events such as graft hypertrophy, delamination, ossification, and contracture, and 5% requiring additional surgery [33]. Furthermore, the long-term durability and qualitative maturation of regenerated tissue remain uncertain. Arthroscopic evaluation and biopsy provide insights into the morphology and quality of ACIs and MACIs [7,8,10,15,19,25,31,32,37]; however, these procedures are invasive. MRI serves as a non-invasive technique for assessing articular cartilage. Magnetic Resonance Observation of Cartilage Repair Tissue (MOCART 2.0) was applied for morphological assessment of ACIs and MACIs [30,32,38,39,40]. However, MOCART 2.0 may provide morphology but not the quality of reparative tissue in ACIs and MACIs. Delayed gadolinium-enhanced MRI for cartilage (dGEMRIC) provides information on glycosaminoglycan (GAG) content [41,42]. However, it requires pre-imaging intravenous contrast medium at twice the routine volume, making it an invasive and potentially risky procedure.

To address these limitations, this study utilized T1*ρ* and T2 mapping as noninvasive, contrast-free quantitative tools to evaluate cartilage repair. Unlike conventional methods, these sequences enable the direct assessment of GAG content and collagen network integrity [43,44,45,46]. The T1*ρ* relaxation time indicates GAG and water content [39,43,44,45], while the T2 relaxation time is related to collagen structure and water [39,44,46], serving as sensitive indicators of tissue quality. Previous research indicates that MRI findings may align with clinical and arthroscopic evaluations, potentially serving as an alternative to invasive procedures. This supports the validity of MRI-based monitoring [47,48,49], although the topic remains contentious. By integrating these with the MOCART 2.0 morphological scale, this study provides a comprehensive multiparametric analysis of pMACI durability that has not been fully established in the existing literature.

We hypothesized that (1) quantitative MRI parameters (T1*ρ* and T2 values) of the repair tissue would correlate with its maturation and durability over time [39,43,44,45], and (2) these non-invasive MRI metrics would demonstrate a significant correlation with clinical scores, potentially serving as reliable alternatives to invasive procedures [47,48,49]. The primary objective of this study was to evaluate the clinical effectiveness and MRI-based durability of pMACI for knee cartilage defects by assessing T1*ρ* and T2 mapping and MOCART 2.0 scores. Furthermore, it aimed to clarify the relationship between quantitative MRI values and clinical outcomes.

## 2. Materials and Methods

### 2.1. Study Design

This was a retrospective cross-sectional study conducted at a single institution.

### 2.2. Patients

Eligible participants for this retrospective cross-sectional analysis underwent pMACI from July 1997 to July 2019, with follow-up ranging from 1 month to 20 years, with an average follow-up of 5.0 years.

The study sample comprised 37 knees from 17 patients (5 males and 12 females) who underwent pMACI at our institution between 1997 and 2019. Within these 37 knees, a total of 50 individual graft implantations were performed. Among the cohort, 12 patients (70.6%) underwent multiple graft procedures within a single knee or repeated evaluation over time, reflecting the long-term durability of pMACI in complex or multifocal cartilage defects. The patient’s symptoms included knee pain, a catching sensation, swelling, and full-thickness articular cartilage defects (Outerbridge grade IV) in the weight-bearing portion of the femoral condyle or patellofemoral joint, as confirmed by MRI. The mean (± standard deviation, SD) ages at pMACI and evaluation were 36.6 ± 15.0 years (range, 16–66 years) and 41.7 ± 15.7 years (range, 17–66 years), respectively (Table 1). The follow-up interval was 5.0 ± 6.7 years (range, 0.1–20). The causes of cartilage defects were trauma (30 knees), OCD (4 knees), and localized OA (3 knees). The mean defect sizes before and after debridement were 7.2 ± 3.4 cm^2^ (range, 2.4–15.0 cm^2^) and 7.3 ± 3.8 cm^2^ (range, 2.4–19.5 cm^2^), respectively. Exclusion criteria included a history of autoimmune disease, hypersensitivity to antibiotics or animal-derived ingredients, or a positive preoperative allergy test for fetal bovine serum and atelocollagen. Additionally, patients for whom imaging tests could not be performed or yielded poor data were excluded from the study. The patients underwent pMACI of the medial or lateral condyle of the femur (20/4) and patella or patellar groove (13/12). This study was approved by the Institutional Committee on Ethics of our University (No. 362), and informed consent was obtained from all the participants.

### 2.3. Matrix-Associated ACI Covered with Autologous Periosteum

pMACI was performed according to the method described by Ochi et al. [25]. Approximately 400 mg of cartilage tissue (400–650 mg) was harvested arthroscopically from non-weight-bearing regions of the knee joint. The cartilage extracellular matrix was removed from the harvested chondrocytes by enzymatic treatment (trypsin and collagenase) [26]. The chondrocytes obtained were embedded in a three-dimensional culture using an atelocollagen gel for 4 weeks in either our institutional laboratory or at Japan Tissue Engineering Co., Ltd., Gamagori, Aichi, Japan. The mean cell concentration in the gel was 3.6 ± 1.4 × 10^6^ counts/cm^3^ (0.7–6.1 × 10^6^ counts/cm^3^). Transplantation was performed via medial or lateral parapatellar arthrotomy using tourniquet control. After inserting the cultured implant into the cartilage defect, the site was covered with a periosteal patch taken from the proximal medial tibia to prevent the transplant from falling out. Finally, the joint capsule, retinaculum, and skin were sutured and closed separately.

### 2.4. Concomitant Surgeries

Instead of pMACI, osteochondral autograft transplantation (OAT) or bone marrow stimulation (MS) was employed to treat minor cartilage defects. Reconstruction was performed to address ligament tears. For meniscal tears, either repair, partial meniscectomy, or meniscal allograft transplantation was conducted. Tibiofemoral malalignment, characterized by a varus or valgus deformity greater than 5°, as well as patellofemoral malalignment indicated by a tibial tuberosity–trochlear groove distance exceeding 15–20 mm, was corrected with osteotomies (Table 1).

### 2.5. Postoperative Rehabilitation

A lightweight brace was used to immobilize the knee for 2 weeks, followed by continuous passive motion of the joint. In patellofemoral lesions, movement was restricted for up to 4 weeks. Partial weight-bearing was introduced 3 weeks post-surgery and gradually increased to full weight-bearing by 8 weeks, depending on the defect site and size, as well as any concomitant issues [29,30,31].

### 2.6. MRI Evaluations

MRI examinations were performed between January 2017 and December 2019 using a 3.0-T MRI scanner (Philips Ingenia Elition 3.0T; Philips Japan, Tokyo, Japan). For MRI examination, we first performed three-dimensional proton-weighted imaging to identify the transplant site. For images requiring T1*ρ* and T2 map generation, slices, including the transplant site, were scanned in the sagittal or coronal plane. Based on the obtained image data, T1*ρ* mapping was generated and measured using “T1calc” provided by Philips Inc., Japan. For T2 mapping, after generating images with the standard MRI scanner application, measurements were performed using ImageJ (version 1.52k; National Institutes of Health, Bethesda, MD, USA). T1*ρ* and T2 relaxation times (T1*ρ* and T2 values) were obtained by measuring the area of the implant site and the adjacent normal cartilage on the same slice for both T1*ρ* and T2 mapping. Global T1*ρ* and T2 indices (T1*ρ* and T2 values in implants divided by the corresponding values in adjacent normal cartilage) were computed [39]. The MOCART 2.0 knee score was used to evaluate implant morphology [50].

### 2.7. Clinical Outcomes

The effectiveness of pMACI was evaluated using the Lysholm Knee Scoring Scale (LKS) and Knee Injury and Osteoarthritis Outcome Score (KOOS) [51]. Treatment failure was defined as a case that required surgical intervention for adverse events at the graft site. Effectiveness was also demonstrated by the number and ratio of cases that achieved the minimal clinically important difference (MCID) between the pre- and post-operative differences, using the cutoff points of LKS and KOOS as defined by Ogura et al. [23], Patient Acceptable Symptomatic State (PASS) using the cutoff points by Chahal et al. [52], and substantial clinical benefit (SCB) using the cutoff points by Ogura et al. [23].

### 2.8. Statistical Analysis

Statistical analyses were performed using linear mixed-effects models (LMM) to evaluate longitudinal changes in clinical scores (LKS and KOOS subscales) and MRI parameters (MOCART 2.0, T1*ρ* index, and T2 index). Random effects included ‘Patient ID’ and ‘Knee ID’ to account for clustering of multiple grafts within the same knee and repeated evaluation of knees within the same patient. Fixed effects included the follow-up duration (years) to estimate annual rates of change (slopes). The correlation between the chronological changes in the clinical scores was analyzed using simple linear regression. Correlations between MRI parameters and clinical outcomes were evaluated using Spearman’s rank correlation coefficient (*Rs*) to account for non-linearity and non-normal distribution. All statistical tests were two-tailed, and a *p*-value < 0.05 was considered statistically significant. All analyses were conducted using (BellCurve for Excel [version 4.09]/R version 4.5.3/SPSS [version 27.0.1.0]).

### 2.9. Sample Size and Power Analysis

A post hoc power analysis was conducted to validate the sufficiency of our sample size (37 knees from 17 patients) for the primary clinical outcomes. Given the observed large effect sizes (Cohen’s *d* = 1.61 for LKS and 2.05 for KOOS total score), the study achieved a statistical power (1-β) of greater than 0.99 at a significance level (α) of 0.05. These results confirm that the study is sufficiently powered to detect clinically meaningful improvements over the 20-year follow-up period, despite the focused number of unique patients.

## 3. Results

### 3.1. Sample Size and Power Analysis

The power analysis indicates that the study had sufficient power to detect signifi-cant longitudinal changes and clinical improvements over the 20-year follow-up peri-od.

### 3.2. Evaluation of Clinical Scores

**Figure 1:**
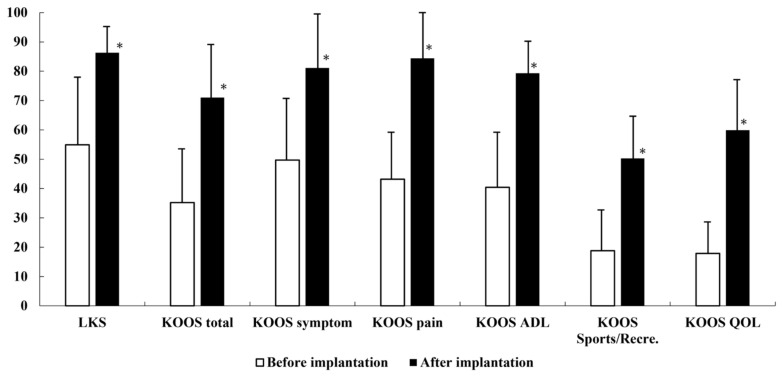
Clinical scores before and after matrix-associated autologous chondrocyte implantation. LKS: Lysholm Knee Scoring Scale, KOOS: Knee Injury and Osteoarthritis Outcome Score, * *p* < 0.001.

All clinical parameters showed significant improvement from preoperative levels to the final follow-up (all *p* < 0.001). The mean LKS increased from 55.6 ± 24.8 (range, 12–94) preoperatively to 86.5 ± 11.2 (range, 61–100) at the final evaluation (Figure 1). Longitudinal analysis using the LMM revealed a slight but significant annual decline of 0.42 points per year (95% CI: −0.68 to −0.16) during the follow-up period.

Similarly, the mean total KOOS increased substantially from 37.8 ± 20.6 (range, 9.8–100) preoperatively to 70.8 ± 18.8 (range, 38.0–97.8) at the final follow-up (Figure 1). Among the subscales, all five categories (Symptoms, Pain, Activities of daily living: ADL, Sport/Recreation: Sport/Rec, and Quality of life: QOL) showed significant improvements (*p* < 0.001; Figure 1). The LMM indicated an annual decrease in total KOOS of 0.38 points (95% CI: −0.62 to −0.14).

**Figure 2:**
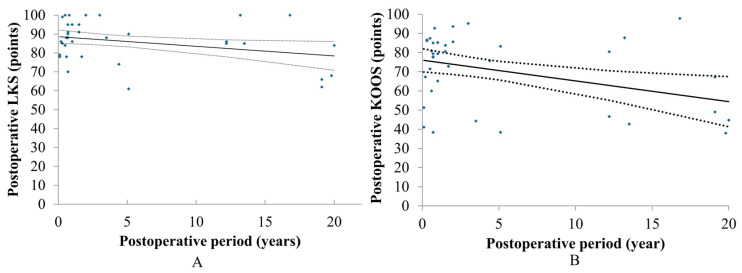
Chronological changes in the postoperative LKS (**A**) and postoperative KOOS (**B**). Correlation with confidence interval set at 95% (dotted lines). LKS: Lysholm Knee Scoring Scale, KOOS: Knee Injury and Osteoarthritis Outcome Score. (**A**). slope = −0.51 points/year, *R*^2^ = 0.09, *p* = 0.030, (**B**). slope = −1.08 points/year, *R*^2^ = 0.14, *p* = 0.008.

Simple linear regression analyses were performed to evaluate the impact of the follow-up interval on clinical outcomes. For LKS, there was a significant but very gradual annual decline (slope = −0.51 points/year, *R*^2^ = 0.09, *p* = 0.030; Figure 2A). The KOOS total score also demonstrated a significant longitudinal decrease (slope = −1.08 points/year, *R*^2^ = 0.14, *p* = 0.008; Figure 2B). Despite these gradual age-related declines, no graft failures necessitating reoperation occurred throughout the observation period.

The LMM-based estimation demonstrated that the clinical benefits of pMACI are highly sustainable over two decades (Table 2). The estimated achievement rates for MCID and PASS on the LKS and KOOS symptom subscales at 2 years postoperatively were 91.8% and 89.2% for the LKS, and 86.5% and 83.8% for the KOOS. At the final follow-up (up to 20 years), these rates remained highly stable at 89.1% and 86.5% for the LKS and 83.7% and 81.1% for the KOOS, respectively, demonstrating that symptomatic improvements are well preserved over the long term. Regarding the SCB in the KOOS Sports/Recreation and QOL subscales, the estimated achievement rates were 64.9% and 67.6% at 2 years postoperatively. At the final follow-up (up to 20 years), these rates remained highly stable at 62.2% and 64.9%, demonstrating that functional improvements in the high-level activities are well preserved over the long term.

### 3.3. Quantitative MRI Evaluation: T1ρ and T2 Mapping

**Figure 3:**
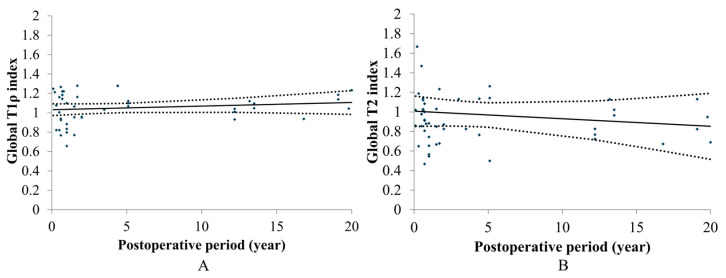
Chronological changes in postoperative global T1*ρ* (**A**) and T2 index (**B**). Correlation with confidence interval set at 95% (dotted lines). (**A**). T1*ρ*: *Rs* = 0.165, *p* = 0.328, (**B**). T2: *Rs* = −0.138, *p* = 0.415.

Postoperative quantitative MRI values showed no significant differences between the pMACI implants and adjacent normal cartilage (T1*ρ*: 44.4 ± 6.4 vs. 42.9 ± 6.7 msec; T2: 124.8 ± 40.2 vs. 135.8 ± 43.1 msec, LKS: *p* = 0.508; KOOS: *p* = 0.562). Longitudinal analysis using LMM indicated that global T1*ρ* and T2 relaxation times remained stable throughout the 20-year follow-up period, with no significant time-dependent changes (*p* = 0.326 for T1*ρ*; *p* = 0.412 for T2). Furthermore, Spearman’s rank correlation analysis confirmed that both T1*ρ* and T2 indices remained stable over time (T1*ρ*: *Rs* = 0.165, *p* = 0.328; T2: *Rs* = −0.138, *p* = 0.415; Figure 3).

Linear regression analyses of biochemical MRI indices demonstrated that the quality of the reparative tissue remained exceptionally stable over the 20-year period. The Global T1*ρ* index showed no significant longitudinal change (slope = −0.0067/year; *p* = 0.0694), with an intercept of 1.08. Similarly, the Global T2 index remained remarkably constant (slope = −0.0079/year; *p* = 0.4385), with the intercept (1.01) and final mean value (0.97 ± 0.44) being nearly identical to unity.

### 3.4. Morphological Assessment: MOCART 2.0

The morphological status of the repair tissue, assessed using the MOCART 2.0 score, showed a significant longitudinal decline over the 20-year period (*p* < 0.001). The mean MOCART score, which was 85.2 ± 10.4 in the early postoperative phase (within 2 years), decreased to 62.4 ± 14.8 at the final follow-up. LMM analysis revealed a significant annual reduction of 1.14 points per year (95% CI: −1.52 to −0.76). This decline was primarily attributed to an increase in subchondral bone abnormalities and surface irregularities in the long-term phase, particularly in cases followed for more than 15 years.

**Figure 4:**
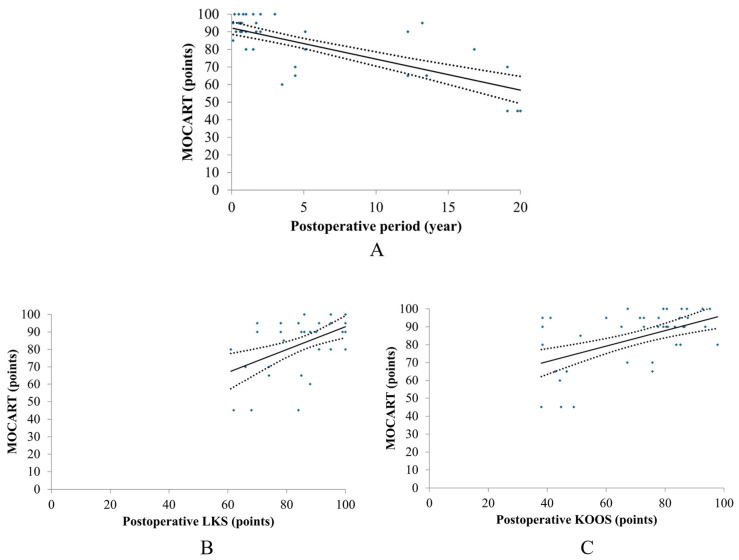
Chronological changes in postoperative MOCART (**A**) and its relationship with postoperative Lysholm knee score (**B**) and KOOS (**C**). Correlation plot with confidence interval set at 95% (dotted lines). MOCART: MR observation of cartilage repair tissue, LKS: Lysholm Knee Scoring Scale, KOOS: Knee Injury and Osteoarthritis Outcome Score. (**A**): *Rs* = −0.421, *p* = 0.009, (**B**): *Rs* = 0.455, *p* < 0.001, (**C**): *Rs* = 0.531, *p* < 0.001.

Linear regression analysis revealed a strong and significant negative correlation between the follow-up interval and the MOCART 2.0 score (*R* = 0.74, *R*^2^ = 0.54, *p* < 0.001). The regression model (*y* = −1.76 *x* + 92.13) indicated a significant annual decline of 1.76 points per year (Figure 4).

This rate of morphological deterioration was more pronounced than the annual declines observed in LKS (−0.51) and KOOS (−1.08). Despite this trend, the high intercept (92.13) confirms excellent initial morphological repair, although subsequent surface irregularities and subchondral bone changes resulted in lower scores in long-term cases beyond 10–15 years.

### 3.5. Effects of Age on Clinical and Radiological Outcomes

We analyzed the correlation between age at surgery and clinical/radiological outcomes. While age showed a moderate negative correlation with the MOCART score (*r* = −0.38, *p* < 0.01), there were no significant correlations between age and postoperative KOOS (*r* = −0.12, *p* = 0.42) or biochemical indices (T1*ρ*: *r* = 0.04, *p* = 0.79; T2: *r* = 0.08, *p* = 0.59).

### 3.6. Effects of Etiology on Clinical and Radiological Outcomes

Clinical and morphological outcomes significantly depend on etiology. OA patients had significantly lower Lysholm and KOOS scores at the 20-year follow-up compared to trauma and OCD groups (*p* < 0.05; Table 3). Similarly, MOCART 2.0 scores were significantly lower in the OA group (*p* < 0.05), reflecting greater morphological deterioration over time.

### 3.7. Effects of Implant Sites on Clinical and Radiological Outcomes

Regarding the graft site (femoral condyles vs. patellofemoral joint: PFJ), there were no significant differences in clinical scores (LKS: *p* = 0.542, KOOS: *p* = 0.489) or MRI parameters (T1*ρ*: *p* = 0.612, T2: *p* = 0.588). However, MOCART 2.0 scores were significantly lower in the PFJ than in the femoral condyles at 20 years (*p* < 0.05), indicating that grafts in the PFJ may be more susceptible to morphological changes due to complex biomechanical stress in this compartment.

### 3.8. Effects of Cartilage Defect Size on Clinical and Radiological Outcomes

Linear mixed model analysis showed no significant correlation between cartilage defect size and evaluated outcomes, including clinical scores (LKS and KOOS; *p* > 0.05) and MRI parameters (T1*ρ*, T2, and MOCART 2.0; *p* > 0.05). These findings suggest pMACI can achieve consistent long-term tissue regeneration and clinical success, even for large defects, if the intra-articular environment is favorable.

## 4. Discussion

This study aimed to evaluate the long-term clinical effectiveness and MRI-based durability of pMACI for knee cartilage defects over up to 20 years. We hypothesized that (1) quantitative MRI parameters (T1*ρ* and T2 indices) would reflect graft maturation and durability over time, and (2) these biochemical metrics would correlate with clinical outcomes. Our main findings demonstrated significant and sustained clinical improvements in LKS and KOOS following pMACI. However, a key finding was the dissociation between biochemical and morphological outcomes: while the repair tissue achieved and maintained biochemical maturation comparable to native cartilage (T1*ρ* and T2 convergence), a gradual morphological deterioration (MOCART 2.0) and a slight decline in clinical scores were observed over the two decades. Notably, clinical outcomes were more closely associated with morphological integrity than with biochemical indices.

Clinical improvement in LKS (30.9 points) in the pMACI was equivalent to that in ACIs (28.96) and MACIs (29.40) reported in the meta-analysis of 47 studies by Nassar et al. [53]. The responders attained the aforementioned optional PASS in 86.5% of LKS, 81.1% of KOOS symptoms, and 86.5% of KOOS pain attributable to pMACI. These results were comparable to or exceeded those achieved with MACI seeding autologous chondrocytes [54]. Furthermore, pMACI facilitated responders in reaching the estimated optional SCB in the KOOS Sports/Recr. and QOL at 62.2% and 64.9%, respectively, and the MCID in the LKS and KOOS symptoms at 89.1% and 83.7%, respectively. The outcomes observed were either comparable to or exceeded those associated with collagen membrane-covered ACI [23]. The efficacy of the periosteal cover in pMACI can be elucidated through the “epiligament theory [34].” Recent studies (2024–2026) have further refined the epiligament theory, highlighting critical biological and morphological differences between the ACL and MCL. While the MCL epiligament is characterized by structural uniformity and coordinated α-smooth muscle actin (α-SMA) activity that supports stable tissue continuity, the ACL epiligament exhibits significant spatial heterogeneity. Specifically, the proximal ACL epiligament shows higher α-SMA expression for tissue remodeling, while the distal portion is enriched with CD34+ progenitor cells, explaining the better healing potential at these ends compared to the relatively inert mid-substance. The superior healing capacity of the MCL over the ACL is attributed to its higher cell density and more robust vascularization within the epiligament [35,36]. In pMACI, the autologous periosteal cover may function as a “surrogate epiligament.” By providing a rich source of regenerative cells and growth factors—similar to the robust environment of the MCL epiligament —it creates a favorable “biological soil” that overcomes the naturally limited healing environment of intra-articular cartilage defects.

In the current study, global T1*ρ* and T2 indices in pMACI varied early postoperatively. Still, they converged to 1.0 over time, resulting in postoperative global T1*ρ* and T2 indices that were not significantly different in the pMACI implants and adjacent normal cartilage.

Numerous studies have evaluated T1*ρ* values following MACIs. A prospective study by Shinohara et al., which included 24 knees undergoing pMACI with follow-up periods extending up to 2 years, reported a decrease in the global T1*ρ* index from 1.5 to 1.4 and then to 1.3 at 6, 12, and 24 months [39]. Matsushita and colleagues found that MACI, using CaReS™ (Arthro Kinetics Biotechnology GmbH, Krems an der Donau, Austria), reduced the global T1*ρ* index from 1.41 to 1.11 and 1.0 at 12, 24, and 48 weeks in nine knees [48]. Baumann-Jungmann et al. investigated T1*ρ* values post-MACI at the patellae, with or without medial patellofemoral ligament reconstruction for patellar instability, over a 2.5-year follow-up period, and observed no significant differences between the groups [55]. The variation in the global T1*ρ* index observed in the early postoperative phase of the current study may be influenced by the concentration of implanted chondrocytes. The convergence of the global T1*ρ* index to 1.0 over 20 years could indicate implant maturation with increasing GAG content, which does not necessarily contradict the findings of previous studies [39,48,55].

Several studies have shown that the T2 values in MACI returned to normal values after implantation. Niethammer et al. reported that matrix-based ACI produced T2 values in the repaired tissue similar to those of normal hyaline cartilage at 36 months post-implantation [56]. Shinohara et al. demonstrated a decrease in the global T2 index from 1.7 to 1.3 and then to 1.2 at 6, 12, and 24 months [39]. Matsushita et al. also reported that the global T2 index decreased from 1.57 to 1.54 and then to 1.14 at 12, 24, and 48 weeks [48]. The present study demonstrated that the global T2 index converged to 1.0 over 20 years, which may align with that of the surrounding normal cartilage. This may reflect the strengthening of collagen construction, suggesting maturation of the pMACI implant.

The current study also revealed that clinical scores were significantly related to the MOCART 2.0 score, indicating that the morphology of the MACI implants, rather than implant quality, was a key factor in determining outcomes. Per recent research, achievement of the PASS, the MCID for KOOS Quality of life, and clinically significant improvements in IKDC scores highly correlate with a more mature MRI appearance of the ACI graft on postoperative MRI, as evidenced by higher MOCART scores [53]. A total of 54 patients who underwent MACI showed that the MOCART 2.0 score influenced long-term KOOS follow-up of 8.1 years [57]. By contrast, Oettl et al. demonstrated that there was no significant correlation between MOCART 2.0 scores and the change in Patient-Reported Outcome Measures (PROMs) following microfracture (N = 37), minced cartilage implantation (N = 22), and ACI (N = 36) over 60 months after surgery, using the IKDC score and Change in Core Outcome Measures and Index [58]. The present study, conducted over 20 years, has the potential to elucidate the relationship between clinical outcomes and MOCART 2.0 scores. The annual decline of 0.45 points in MOCART scores suggests a subtle, inevitable structural remodeling. However, the remarkable stability of PASS achievement rates (with declines of less than 3% over 20 years) confirms that these radiological changes are clinically negligible. This ‘decoupling’ of imaging and function reinforces the role of pMACI as a durable gold standard for large cartilage defects. Unlike microfracture, which often shows a decline in clinical outcomes after 5 to 10 years due to the limited durability of fibrocartilage [53,59], our pMACI results demonstrated sustained clinical benefits and biochemical tissue integrity for up to 20 years.

A clear dissociation was shown between biochemical and morphological outcomes: while the biochemical properties of the pMACI implants—as reflected by indices converging toward 1.0—were sustained long term, the morphological scores (MOCART 2.0) showed a significant time-dependent decrease (*p* < 0.001). Furthermore, no significant correlations were identified between these MRI indices and postoperative clinical scores (LKS: *p* = 0.508; KOOS: *p* = 0.562), suggesting that structural integrity and joint-wide aging factors, rather than biochemical tissue quality alone, may primarily determine late-stage clinical fluctuations. This suggested that the initial biochemical maturation does not fully protect against long-term degenerative changes.

Although the heterogeneity of our cohort precluded a formal Kaplan–Meier survival analysis for each etiology, no cases of clinical failure requiring reoperation were observed in this series. This suggests a high cumulative survival rate for pMACI grafts for up to 20 years. Future prospective studies with larger, more homogeneous cohorts will be essential to define the precise survival curve of these implants.

The results on the effects of age on clinical and radiologic outcomes suggest that while morphological repair may be slightly affected by aging [60], pMACI can achieve high-quality biochemical tissue maturation and satisfactory clinical outcomes regardless of patient age.

The poorer outcomes observed in patients with early osteoarthritis (OA) suggest that graft survival is influenced not only by the quality of the regenerated tissue itself but also by the broader intra-articular environment. From a clinical perspective, this indicates a clear limitation of pMACI in a degenerative environment. Therefore, clinicians must prioritize strict patient selection and consider addressing the joint environment—such as correcting malalignment or stabilizing the joint [61]—prior to or alongside pMACI. Ultimately, ensuring a favorable “biological soil” is as crucial as the “seed” of the cultured cartilage for achieving twenty-year durability.

Comparison of the implant sites indicates that the biochemical maturation of the repair tissue into a hyaline-like matrix proceeds consistently regardless of the anatomical site or its mechanical environment. These findings statistically support the conclusion that pMACI consistently provides biological repair and patient satisfaction, even when morphological scores vary across disease backgrounds. However, the MOCART 2.0 scores were significantly lower in the patellofemoral joint compared to the femoral condyles at 20 years (*p* < 0.05), indicating that grafts in the PFJ may be more susceptible to morphological changes due to the complex biomechanical stress in this compartment [62].

The current study has some limitations. First, the sample size was relatively small (37 knees from 17 patients), which may limit the generalizability of our findings, particularly in subgroup analyses of rare etiologies such as OCD. However, the longitudinal design and the application of mixed-effects models enabled a robust assessment of temporal changes while accounting for the dependence among observations. Future multi-center studies with larger cohorts are warranted to confirm our results on long-term durability. Secondly, there was a potential of losing control or reference for comparing pMACI, as many concomitant surgeries and different sites and pathologies have been treated, which has reduced the ability to analyze subgroups. A collagen membrane might improve the outcomes of pMACI by reducing the periosteum-related issues, such as hypertrophy, delamination, or ossification [33]. A collagen membrane-covered MACI might, therefore, result in changes in outcomes and MRI-based durability compared with the pMACI.

## 5. Conclusions

The present study suggested that pMACI may be a clinically effective, durable treatment for knee cartilage defects, with graft quality and patient-reported outcomes potentially maintained for up to 20 years. Our findings potentially suggest that while the reparative tissue may achieve biochemical maturation comparable to native cartilage, morphological deterioration could occur over two decades due to joint aging and degenerative changes. Given the sustained clinical benefits, pMACI remains a highly suitable option for younger, active patients with traumatic defects. However, the observed late-stage morphological changes highlight the necessity for long-term clinical and MRI monitoring beyond the second decade to assess potential degenerative progression.

## Figures and Tables

**Table 1:** Demographic Data.

**Age [years] (range)**	
At pMACI	36.6 ± 15.0 (16–66)
At evaluation	41.7 ± 15.7 (17–66)
**Sex**	
Male	5
Female	12
**Follow-up Interval (years)**	5.0 ± 6.7 (0.1–20.0)
**Disease**	
Traumatic cartilage defects	30
Osteochondritis dissecans	4
Localized osteoarthritis	3
**Mean lesion size [cm^2^/knee] (range)**	
Before debridement	7.2 ± 3.4 (2.4–15.0)
After debridement	7.3 ± 3.8 (2.4–19.5)
**Number of grafts**	
Single	19
Multiple	18
**Defect location**	
Medial femoral condyle	20
Lateral femoral condyle	4
Patella	13
Patellar groove	12
**Concomitant injuries**	
Meniscal tear	6
Cruciate ligament injury	5
Femorotibial joint malalignment	4
Patellofemoral joint malalignment	10
**Concomitant surgeries**	
High tibial osteotomy	4
Tibial tubercle osteotomy	1
MPFL reconstruction	10
ACL reconstruction	4
PCL reconstruction	1
Meniscal suture	6
OAT	16
Bone marrow stimulation	3
Meniscal allograft transplantation	2

pMACI: matrix-associated autologous chondrocyte implantation covered with periosteal flap, ACL or PCL: anterior or posterior cruciate ligament, MPFL: medial patellofemoral ligament, OAT: osteochondral autograft transplantation.

**Table 2:** Estimated clinical success rates at 2 years and final follow-up based on linear mixed-effects models.

Outcome Measure	Timepoint	MCID (%)	PASS (%)	SCB (%)
LKS	2-Year	91.8	89.2	78.4
	Final	89.1	86.5	75.7
KOOS Symptoms	2-Year	86.5	83.8	75.7
	Final	83.7	81.1	73
KOOS Pain	2-Year	78.4	89.2	54.1
	Final	75.7	86.5	51.4
KOOS ADL	2-Year	73	70.3	70.3
	Final	70.3	67.6	67.6
KOOS Sports/Recr.	2-Year	64.9	51.4	64.9
	Final	62.2	48.6	62.2
KOOS QOL	2-Year	70.3	64.9	67.6
	Final	67.6	62.2	64.9

LKS: Lysholm Knee Scoring Scale, KOOS: Knee Injury and Osteoarthritis Outcome Score, MOCART: Magnetic Resonance Observation of Cartilage Repair Tissue. ADL: activities of daily living, Recr.: recreation, QOL: quality of life. All values represent estimated percentages of patients achieving the thresholds, calculated using linear mixed-effects models. Final follow-up indicates the estimated status at the mean maximum observation period (up to 20 years).

**Table 3:** The effect of the etiology on the clinical scores and radiographic evaluations at 20-year follow-up based on linear mixed-effects models.

Estimated Outcomes	Trauma	OCD	OA	95% CI (OA)	*p* Value
LKS	88.2 ± 10.5	87.5 ± 11.2	74.1 ± 14.8	[68.5, 79.7]	*p* < 0.05
KOOS Overall	78.5 ± 12.1	76.9 ± 13.5	58.2 ± 16.4	[52.1, 64.3]	*p* < 0.05
T1*ρ* (ms)	42.1 ± 4.5	43.5 ± 5.2	44.2 ± 6.1	[41.2, 46.5]	*p* = 0.326
T2 (ms)	38.2 ± 3.8	39.1 ± 4.5	40.5 ± 5.2	[37.5, 41.8]	*p* = 0.412
MOCART 2.0	68.5 ± 15.2	65.2 ± 16.8	48.6 ± 19.5	[41.2, 56.0]	*p* < 0.05

LKS: Lysholm Knee Scoring Scale, KOOS: Knee Injury and Osteoarthritis Outcome Score, MOCART: Magnetic Resonance Observation of Cartilage Repair Tissue, OCD: Osteochondritis Dissecans, OA: Osteoarthritis. *p*-value: Comparison of the values of OA with those of Trauma and OCD.

## Data Availability

Raw data were generated at Shimane University. Derived data supporting the findings of this study are available from the corresponding author T. Tadenuma on request.

## Abbreviations

The following abbreviations are used in this manuscript:

pMACI	Matrix-associated Autologous Chondrocyte Implantation with Periosteal flap
OCD	osteochondritis dissecans
OA	osteoarthritis
MPFL	medial patellofemoral ligament
ACL	anterior cruciate ligament
MCL	medial collateral ligament
OAT	osteochondral autograft transplantation
MCID	minimal clinically important difference
PASS	Patient Acceptable Symptomatic State
SCB	substantial clinical benefit
LKS	Lysholm Knee Scoring Scale
KOOS	Knee Injury and Osteoarthritis Outcome Score
MOCART	MR observation of cartilage repair tissue
